# UPLC–MS/MS Identification of Sterol Sulfates in Marine Diatoms

**DOI:** 10.3390/md17010010

**Published:** 2018-12-24

**Authors:** Genoveffa Nuzzo, Carmela Gallo, Giuliana d’Ippolito, Emiliano Manzo, Nadia Ruocco, Ennio Russo, Ylenia Carotenuto, Maria Costantini, Valerio Zupo, Angela Sardo, Angelo Fontana

**Affiliations:** 1 Bio-Organic Chemistry Unit, Institute of Biomolecular Chemistry CNR, Via Campi Flegrei 34, Pozzuoli, 80078 Naples, Italy; carmen.gallo@icb.cnr.it (C.G.); gdippolito@icb.cnr.it (G.d.I.); emanzo@icb.cnr.it (E.M.); nadia.ruocco@gmail.com (N.R.); angela.sardo@icb.cnr.it (A.S.); afontana@icb.cnr.it (A.F.); 2 Department of Marine Biotechnology, Stazione Zoologica Anton Dohrn, 80121 Naples, Italy; mcosta@szn.it (M.C.); vzupo@szn.it (V.Z.); 3 Department of Integrative Marine Ecology, Stazione Zoologica Anton Dohrn, 80121 Napoli, Italy; ennio.erre@gmail.com (E.R.); ylenia@szn.it (Y.C.)

**Keywords:** terpene, sterol, diatom, dinoflagellate, sulfotransferase, marine organism, natural product, secondary metabolite, mass spectrometry, chromatography

## Abstract

Diatoms are unicellular eukaryotic organisms that play a key ecological and biogeochemical role in oceans as major primary producers. Recently, these microalgae have also attracted interest as a promising source of functional products with widespread relevance. Progress in the knowledge of cell and molecular biology of diatoms is envisaged as a key step to understanding regulation of their life cycle in marine environments as well as facilitating their full and profitable exploitation by biotechnological platforms. Recently, we identified sterol sulfates (StS) as regulatory molecules of cell death in the diatom *Skeletonema marinoi*. As these compounds may have a general role in diatom physiology and chemical signals in aquatic systems, we investigated a suitable tool for their analysis in laboratory and field samples. Herein, we describe a sensitive, fast, and efficient ultra performance liquid chromatography–mass spectrometry (UPLC–MS) method for qualitative and quantitative analysis of StS from crude extract of diatoms and other microalgae. The method was applied to 13 different strains of our collection of marine protists. This first study suggested a species-specific distribution of StS and identified the sulfated derivatives of 24-methylene cholesterol and 24-methyl cholesterol as the most common members in diatoms.

## 1. Introduction

Diatoms are a highly diverse and abundant group of phytoplankton. As photosynthetic organisms, they contribute to global primary productivity and have a central role in biogeochemical cycling of carbon, nitrogen and silica [1,2,3]. Furthermore, diatoms are being investigated for the carbon neutral synthesis of biofuels and as a source of functional products, including pharmaceuticals, health foods, and nanomaterials [4].

Although phytoplankton is traditionally composed of passive drifters, many recent reports have demonstrated that these eukaryotic microbes employ complex mechanisms to sense changes in environmental cues and activate chemical-based defense [5]. These mechanisms have been mainly associated with the synthesis of chemical signals (e.g., infochemicals) triggered by biotic and abiotic interactions. For example, when diatoms are subjected to grazing or nutrient stress, cells can rapidly induce the biosynthesis of lipoxygenase products responsible for the production of apoptotic polyunsaturated aldehydes (PUAs) and nonvolatile oxylipins [6,7,8,9,10,11,12].

Continuing our research on ecologically and physiologically relevant compounds in marine habitats [11,12,13,14,15,16], we recently reported that the production of sterol sulfates (StS) was linked to growth control and cell death in the marine bloom-forming diatom *Skeletonema marinoi* [17]. In this microalga, we identified three different StS (cholesterol, dihydrobrassicasterol, and β-sitosterol sulfate) whose concentrations changed along the growth curve and induced apoptosis-like mechanism in a dose-dependent manner, suggesting that these molecules play a role as intracellular mediators in physiological and ecological functions of diatoms. Programmed cell death is considered as one of the major causes for control of algal blooms; thus StS alone or in synergy with other molecules might determine the duration of the growth curve of these microalgae and affect the seasonal succession of phytoplankton species in marine environments. On the other hand, inhibition of StS synthesis increases the number of algal cells [17] and is a potential mechanism for multiplying algal biomass to be used in biotechnological applications or as a starting material for the extraction of functional compounds. In this view, a fast and sensitive liquid chromatography–mass spectrometry (LC–MS) method for the identification and quantification of StS may represent a suitable analytical technique to support studies in monitoring seasonal dynamics of diatom populations as well as find direct and general application in the development of diatom-based platforms for biotech applications. For this purpose, we present herein a new sensitive and fast ultra-performance liquid chromatography–electrospray ionization tandem mass spectrometry (UPLC–ESI-MS/MS) for StS investigation in microalgae and other biological samples. The method was set up using synthetic StS synthesized by sulfation of commercially available sterols. The reliability of the analytical approach was validated on *S. marinoi* and confirmed on marine photoautotrophic protists, including dinoflagellates and other diatoms.

## 2. Results

**Figure 1:**
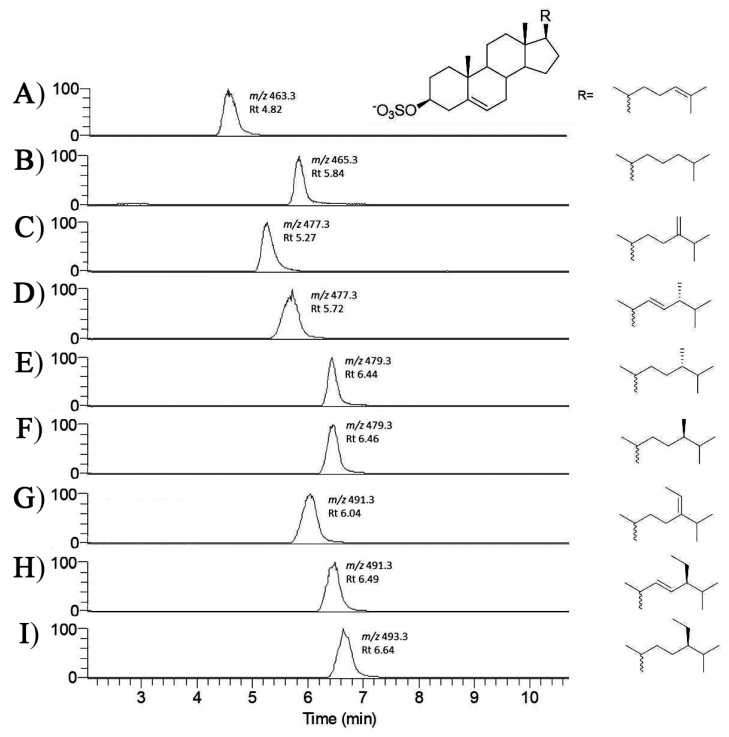
LC–MS chromatograms of synthetic standards of sterol sulfates under the analytical conditions described in this work. (**A**) desmosterol sulfate; (**B**) cholesterol sulfate; (**C**) 24-methylene cholesterol sulfate; (**D**) brassicasterol sulfate; (**E**) dihydrobrassicasterol sulfate; (**F**) campesterol sulfate; (**G**) fucosterol sulfate; (H) stigmasterol sulfate; (**I**) β-sitosterol sulfate. Values near each peak represent retention time and molecular mass as obtained by ion extract mode.

UPLC–ESI-MS/MS was used to identify StS composition directly from crude organic extracts. The chromatographic method was based on a biphenyl UPLC column and a gradient of MeOH in water that allowed the elution of StS as a sharp peak in the range between 4.5 and 7 min over a total run time of 20 min (Figure 1). Chromatographic conditions were optimized in several trials to achieve good resolution and symmetrical peak shapes. The UPLC system was coupled to a high-resolution Q-Exactive benchtop hybrid quadrupole-orbitrap mass spectrometer equipped with a high-resolution electrospray ionization (HR-ESI) probe operating in negative ionization mode. Combining the advantages of high resolution (70 k), accurate mass measurements (typically below 2 ppm), and tandem mass analysis, the method permitted the identification of StS at very low concentrations with a detection limit of 20 pg.

**Scheme 1:**
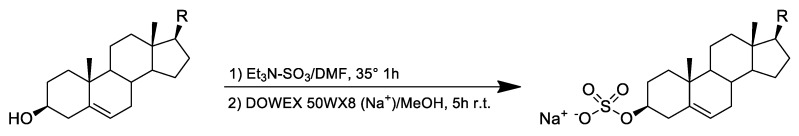
Synthesis of sterol sulfates by direct sulfation of commercially available sterols.

Except for cholesterol sulfate, other sterol sulfates were not easily available in the market. For this reason, we built a small library of StS containing synthetic standards prepared by sulfation of the commercial sterols 24-methylene cholesterol, desmosterol, campesterol, brassicasterol, dihydrobrassicasterol, stigmasterol, fucosterol, and β-sitosterol. As reported in Scheme 1, sulfation was carried out by reaction of 1 mg of each sterol with triethylamine–sulfur trioxide complex in *N*,*N*-dimethylformamide as polar aprotic solvent. To obtain the sodium salt, the reaction mixture was passed through the cation exchange resin DOWEX 50WX8 (sodium form, Na^+^) in MeOH [18]. The synthesis was quantitative and, after purification by silica gel followed by reversed-phase high-performance liquid chromatography (RP-HPLC), recovery of the sulfated products gave an average yield of 65%.

**Figure 2:**
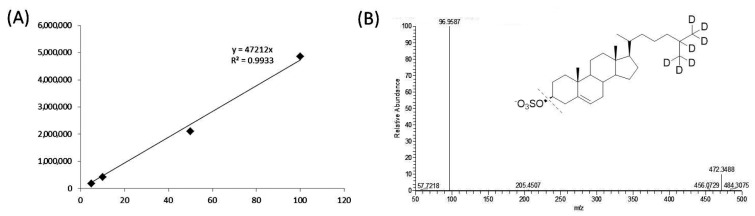
Electrospray ionization mass spectrometry (ESI-MS) response to sterol sulfates. (**A**) Calibration curve and (**B**) diagnostic loss of sulfate from sterol sulfates as exemplified by fragmentation of deuterated cholesterol sulfate used as internal standard.

In order to establish the recovery yield of StS and to perform quantitative analysis of natural products, commercially available [25,26,26,26,27,27,27-D7]-cholesterol-sulfate (CHOS-d) was added as internal standard to the microalgal pellet prior to extraction. The calibration curve for the ratio of peak area–concentration of CHOS-d showed a linear relationship over a concentration range of 1 to 100 ng/mL, that is in the range of the physiological occurrence of StS in the microalgal samples [17,18]. The regression equation was *y* = 4721*x*, and the correlation coefficient (*r*^2^) was 0.9933, indicating good linearity (Figure 2). Deviation from the expected concentration ranged from 97.4% to 102.0%, and the coefficients of variation were all within 5%. Similar results were also observed with other synthetic sterol sulfates (data not shown), thus corroborating the reliability of the method performance. The characteristic fragment at *m*/*z* 97 in the MS/MS spectra was associated with the loss of the sulfate group and was used as a diagnostic marker for the unambiguous identification of StS in complex mixtures.

**Figure 3:**
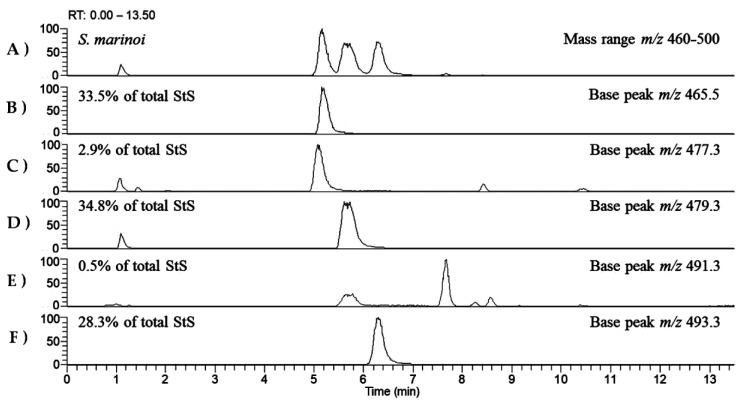
Liquid chromatography–tandem mass spectrometry (LC–MS/MS) analysis of sterol sulfates in *S. marinoi*. (**A**) Total ion current chromatogram in the range between 460 and 500 *m*/*z*; (**B**) cholesterol sulfate; (**C**) 24-methylene cholesterol sulfate; (**D**) dihydrobrassicasterol sulfate; (**E**) fucosterol sulfate; (**F**) β-sitosterol sulfate. Percentage of each sterol sulfate is reported on the left part of each spectrum, whereas the corresponding molecular ion is shown on the right.

The above method was first applied for the analysis of *S. marinoi* as proof of concept and then tested on other eight diatom strains including five benthic (*Nanofrustulum shiloi*, *Cylindrotheca closterium*, *Cocconeis scutellum*, *Phaeodactylum tricornutum*, and *Diploneis* sp.) and three planktonic species (*Cyclotella cryptica*, *Thalassiosira rotula*, and *Thalassiosira pseudonana*). In addition, we also assessed, for the first time, the presence of StS in dinoflagellates, namely, *Heterocapsa* sp., *Scripsiella* sp., *Amphidinium massarti*, and *Amphidinium carterae*, which are known to biosynthesize a specific family of sterols known as dinosterols. In agreement with our previous report [17], the analysis of *S. marinoi* confirmed the presence of cholesterol, dihydrobrassicasterol, and β-sitosterol sulfates and revealed the presence of two additional minor compounds—24-methylene cholesterol sulfate and fucosterol sulfate—representing 2.9 and 0.5% of the total StS content, respectively (Figure 3). Among the other protists, all diatom species (*N. shiloi*, *C. closterium*, *C. scutellum*, *Diploneis* sp., *T. rotula*, *P. tricornutum*, *T. pseudonana*, and *C. cryptica*) showed significant level of sterol sulfates, whereas these compounds were not detectable in dinoflagellates (*Heterocapsa* sp., *Scripsiella* sp., *A. massarti*, and *A. carterae*).

In agreement with the literature about diatom sterols [19], UPLC–MS of diatom extracts revealed that the most common metabolites were the sulfated derivatives of 24-methylene cholesterol and 24-methyl cholesterol. Moreover, like in plants, StS were generally present in diatoms as mixtures of more components that mostly differed in the substitution at C-24 of the side chain by methyl, ethyl, and propyl residues. The good correlation between intraspecific composition of the pools of sterols and sterol sulfates shows that sulfation can occur without selection of the substrates. This may imply the occurrence of a single class of sulfotransferase for the sulfation of this family of compounds [18]. Interestingly, we detected single products in only two species, namely, *C. closterium* and *Diploneis* sp., that contained desmosterol sulfate and brassicasterol sulfate, respectively (Table 1; Supplementary Table S1).

To confirm the assignment of unknown StS, due to the little difference in the retention time between structural isomers with the same *m*/*z*, analyses in coelution with presumed standard were also performed (data not shown). The LC–MS data do not provide information on the configuration of the substituents at C-24, thus the method cannot be applied to distinguish stereoisomers, such as dihydrobrassicasterol and campesterol sulfate, that differ only in the α- or β-orientation of the methyl group (Table 1). For *S. marinoi* and *C. cryptica*, identification of dihydrobrassicasterol sulfate compound was achieved after purification and structure elucidation by NMR of the natural product (Table 1). Besides this, the analysis of different microalgae delineated a species-specific composition of StS, thus suggesting a good chemotaxonomic and biochemical support.

The presence of CHOS-d as internal standard also allows quantitation and assessment of recovery yields. For this reason, in addition to the qualitative analysis, the UPLC–MS method is suitable for the quantification of sterol sulfates in natural samples. As proof of concept, Table 2 reports the total amount of StS in the cluster composed of the benthic diatoms *C. closterium*, *N. shiloi*, *C. scutellum*, and *Diploneis* sp. In order to compare the different species, concentration of StS in micrograms were normalized per milligram of carbon as measured by CHNS elemental analysis on dry biomass. It is worth noting that StS are minor metabolites, and the amount produced is steadily increased from the beginning to the end of the growth curve [17]. For this quantitative study, the species subjected to analysis were obtained from cell pellets harvested during the declining phase.

## 3. Discussion

Sterol sulfates are natural products that, before our report in diatoms [17], have been described in many marine invertebrates [20,21,22,23,24,25,26,27,28]. The molecules represent a class of chemical mediators biosynthesized by transfer of a sulfo moiety (SO_3_H^−^) to the secondary hydroxyl function at C-3 of sterols. The reaction is catalyzed by a broad family of enzymes named sulfotransferases that have a recognized role in the metabolism of many organisms, including mammalians and plants [18]. The conversion of sterols to StS gives new biological functions and changes the chemophysical properties of the organic compounds. However, although sulfation has also been used as derivatization for the detection and quantification of apolar sterols by LC–MS analysis [29], all methods available for quantification suffer from several technical limitations (ionization, chromatographic resolution, sensibility, and reproducibility). Thus, the development of a new UPLC–MS method for the study of StS appears useful not only in the study of the physiology of marine diatoms but even in other research fields including biomedical applications where qualitative and quantitative analysis of these metabolites is important as potential disease biomarkers [30,31,32]. Although cholesterol 3-sulfate, at low levels, can be the main sulfolipids in many cell types, it has been reported to have a variety of signaling and regulatory functions toward many aspects of lipid metabolism, inflammatory responses, and cell proliferation through its actions on nuclear receptors [33]. To the best of our knowledge, even in the most comprehensive LC–MS methods proposed to date, analysis requires complex solvent mixtures and the use of additives for pH control to improve peak shape, resolution, and ionization of the charged lipids [32]. On the other hand, the method reported here showed a satisfactory chromatographic resolution, sensitivity, and ionization of this molecules by the use of a elution gradient of MeOH in water, thus drastically simplifying the procedure of solvent preparation and elution of the metabolites.

The study of the levels of StS in the two major groups of marine photoautotrophic eukaryotic microbes underlines their presence in diatoms, with differences in concentration and composition among the different species, as well as suggests their absence in dinoflagellates. This is an intriguing aspect that deserves further investigation. In fact, dinoflagellates contain a very specific pool of sterols, named dinosterol and its analogs, which are chemically characterized by the presence of a methyl group at C-4. The absence of the corresponding sulfates may indicate the absence of specific sulfotransferases as well as the occurrence of biochemical mechanisms that can replace the physiological role of StS in these eukaryotic cells.

In conclusion, we have developed a simple and robust LC–MS/MS method that is suitable but not limited to the qualitative and quantitative analysis of StS in marine samples. The platform allows the study of a large number of samples in a relatively short time. In particular, the method has been designed for the analysis of field samples and, in our opinion, can provide a very useful tool to study marine phytoplankton communities.

## 4. Materials and Methods

### 4.1. General

Methanol and water, LC/MS grade, were from MercK (Darmstadt, Germany). [25,26,26,26,27,27,27-D7]-cholesterol sulfate standard (CHOS-d) and cholesterol sulfate were purchased from Sigma-Aldrich (Milan, Italy). Reference sterol sulfates were synthesized in-house by chemical derivatization of sterols (24-methylene cholesterol, desmosterol, campesterol, brassicasterol, dihydrobrassicasterol, stigmasterol, fucosterol, and β-sitosterol) that were purchased from Sigma-Aldrich (Milan, Italy). HPLC for synthetic StS standard purification were performed on a JASCO system (PU-2089 Plus quaternary gradient pump equipped with a MD-2018 Plus photodiode array detector and Sedex 85 high-sensitivity LT-ELS detector). UPLC–MS analysis was performed on Q-Exactive hybrid quadrupole-orbitrap mass spectrometer (Thermo Scientific, Waltham, MA, USA) equipped with an Infinity 1290 UHPLC System (Agilent Technologies, Santa Clara, CA, USA).

### 4.2. Chemical Derivatization of Sterols to Sterol Sulfates

0.01 mmol of each sterol was dissolved in anhydrous *N*,*N*-dimethylformamide (0.5 mL), and sulfur trioxide-triethylaminecomplex (0.04 mmol) was added to the solution. The reaction mixture was stirred at 35 °C under argon atmosphere for 1 h. After quenching by 0.2 mL MilliQ water, the mixture was purified on silica gel column to remove the excess of triethylamine–sulfur trioxide. The resulting StS was dissolved in MeOH and mixed with 0.5 g Dowex50WX8 in the sodium form. After stirring for 5 h at room temperature, the sample was filtrated on paper, and the process was repeated on the solution. Sodium salt of StS was dried by rotary evaporator as yellowish powder. Sample purification was finally achieved on analytical reversed-phase column (Phenomenex, C-18 Luna 4.6 × 250 mm, 100 Å) using a gradient of CH_3_OH/H_2_O in agreement with Reference [17]. Elution was carried out by a flow rate of 1 mL min^−1^, and StS were detected by an evaporative light-scattering detector (evaporation temperature 45 °C, nebulization temperature 60 °C, N_2_ flow 1.4 mL min^−1^). Pure products were suspended in MeOH and diluted to obtain a MS sample of 5 µg/mL. For the analysis, 5 μL of this solution was injected under the LC–MS conditions described below.

### 4.3. Microalgal Material

*Skeletonema marinoi* (CCMP 2092 purchased from Bigelow Laboratories) (CCMP331) was used to validate the method, whereas five benthonic (*Nanofrustulum shiloi*, *Cylindrotheca closterium*, *Cocconeis scutellum*, *Diploneis* sp., and *Phaeodactylum tricornutum*), three planktonic diatoms (*Cyclotella. cryptica*, *Thalassiosira rotula*, and *Thalassiosira pseudonana*), and four dinoflagellates (*Heterocapsa* sp., *Scripsiella* sp., *Amphidinium massarti*, and *Amphidinium carterae*) were analyzed for identification of StS.

*S. marinoi* was grown in f/2 medium [34] at 100 μmol (photons) m^−2^ s^−1^, at 20 ± 2 °C, with 14:10 h (light/dark) photoperiod and pH 7 by 50 mM Mops. *C. cryptica*, *T. rotula*, and *T. pseudonana* were grown in f/2 medium [34] at 200 μmol (photons) m^−2^ s^−1^, at 20 ± 2 °C, with 14:10 h (light/dark) photoperiod and gently bubbled with steril air, whereas *P. tricornutum* was grown in f/2 in the same condition of light and temperature reported for the diatoms described above.

*N. shiloi*, *C. closterium*, *C. scutellum*, and *Diploneis* sp. were grown in 17-cm glass Petri dish [35] plates in f/2 medium at 140 μmol m^−2^ s^−1^ and 18 ± 1.0 °C, with a 12:12 h (light/dark) photoperiod.

Dinoflagellates (*Heterocapsa* sp., *Scripsiella* sp., *A. massarti*, and *A. carterae*) were cultured in K medium at 22.0 ± 0.5 °C, under a 14:10 h light/dark regime and at 100 µmol m^−2^ s^−1^ in sterile 1.8 L glass Fernbach bottles, each containing 1 L of culture.

All diatoms were harvested at late exponential-early stationary phase by centrifugation in a swing-out Allegra X12R (Beckman Coulter Inc., Palo Alto, CA, USA) at 2300 *g*, 4 °C, for 10 min 1800 *g*, 4 °C). Algal pellets were immediately frozen in liquid nitrogen and stored at −80 °C until chemical analyses. Cell growth (cells mL^−1^) was estimated daily using a Bürker counting chamber (Merck, Leuven, Belgium) (depth 0.100 mm) under an inverted microscope.

### 4.4. Sample Preparation for LC/MS Analysis

For each microalgae, wet cell pellet (~50 mg) was suspended with 250 μL of MeOH. After sonication for 5 min, the sample was centrifuged at 1000 *g* for 10 min at 4 °C, and the organic phase was recovered. The pellet was re-extracted with MeOH, the supernatant was combined in vial, and the organic solvent was removed under nitrogen stream. Each extract was finally suspended in LC–MS-grade MeOH and diluted to obtain a MS sample of 50 µg/mL of which 5 μL were injected for LC–MS analyses. For quantitative analysis, 1 μg of d7-cholesterol sulfate dissolved in MeOH was added to each 10 mg of diatom pellet (wet biomass) before extraction. For each run, peaks of sterol sulfates were extract to verify the presence of the diagnostic fragmentation *m*/*z* 97 in the MS/MS spectra due to the loss of the sulfate group and to compare the retention time to the deuterated standard (*m*/*z* 472.3; see Supplementary Table S1). Experiments were performed in triplicate.

### 4.5. Ultra Performance Liquid Chromatography/High Resolution Mass Spectrometry (UPLC/HRMS)

Chromatographic separations were achieved on Kinetex Biphenyl 2.6 μm, 150 × 2.1 mm column (Phenomenex, Italy), at 28 °C. Eluent A was water, and eluent B was MeOH. After 2 min of isocratic condition with 70% of B, the elution program consisted of a gradient from 70 to 85% B in 8 min, then to 100% B in 5 min, and holding at 100% B for 5 min more. A post-run equilibration step of 5 min was included prior to each analysis. The flow rate was 0.3 mL·min^−1^. The injection volume was 5 μL, and the autosampler was maintained at 10 °C. MS analyses were carried out on Q-Exactive hybrid quadrupole-orbitrap mass spectrometer in ESI negative mode. Source parameters were as follows: spray voltage of 3.0 kV, capillary temperature of 320 °C, S-lens RF level of 45, auxiliary gas temperature of 320 °C, sheath gas flow rate of 40, auxiliary gas flow rate of 30. Full MS scans were acquired over the range 200–600 with a mass resolution of 70,000. The target value (AGC) was 1 × 10^6^ and the maximum allowed accumulation time (IT) was 100 ms. For the data-dependent MS/MS (ddMS2) analyses, a Top 10 method was used. The 10 most intense peaks were selected for fragmentation with a stepped normalized energy of 20–25–30 eV. AGC was 1 × 10^5^ with IT 75 ms and 17,500 mass resolution.

Linearity, calibration curve, and limit of detection were evaluated on [25,26,26,26,27,27,27-D7]-cholesterol sulfate standard (CHOS-d). The calibration curve was obtained by spiking increasing concentrations of standard solution prepared in MeOH, including the following points: 100, 50, 10, 5, and 1 ng/mL. Each sample was analyzed in triplicate.

## Figures and Tables

**Table 1:** Distribution of sterol sulfates in the diatom species under analysis in this study. The method does not allow to discriminate among epimers.

Compounds	Molecular Formula	Calculated MS	Measured *m*/*z*	Species Distribution
Desmosterol sulfate	C_27_H_43_O_4_S^−^	463.2887	463.2896	*C. closterium* *P. tricornutum*
Cholesterol sulfate	C_27_H_45_O_4_S^−^	465.3044	465.3047	*S. marinoi*
24-methylene cholesterol sulfate	C_28_H_45_O_4_S^−^	477.3044	477.3043	*S. marinoi* *C. cryptica* *C. scutellum* *P. tricornutum* *T. pseudonana* *T. rotula*
Brassicasterol sulfate (24β-methyl-22-dehydrocholesterol)	C_28_H_45_O_4_S^−^	477.3044	477.3052	*N. shiloi* *C. scutellum* *Diploneis* sp.
24-methyl cholesterol sulfate	C_28_H_47_O_4_S^−^	479.3200	479.3205	*S. marinoi* ^1^ *C. Cryptica* ^1^ *T. pseudonana* *T. rotula*
Fucosterol sulfate	C_29_H_47_O_4_S^−^	491.3200	491.3201	*S. marinoi* *N. shiloi*
β-Sitosterol sulfate (24β-ethyl cholesterol)	C_29_H_49_O_4_S^−^	493.3357	493.3361	*S. marinoi*

^1^ Dihydrobrassicasterol sulfate, as characterized after purification and NMR analysis of the natural product.

**Table 2:** Quantitative analysis of sterol sulfates in the benthic diatoms *Cylindrotheca closterium*, *Nanofrustulum shiloi*, *Cocconeis scutellum*, and *Diploneis* sp. Sterol sulfate content is expressed as µg/mg of carbon (dry weight). Each sample was analyzed in triplicate. Means ± standard error is reported.

Diatom Species	Compounds	µg StS/mg C
*C. closterium*	Desmosterol sulfate	26.21 ± 2.6
	Total StS	26.21 ± 2.6
*N. shiloi*	Brassicasterol sulfate	5.94 ± 1.0
	Fucosterol sulfate	60.69 ± 7.3
	Total StS	66.63 ± 5.2
*C. scutellum*	24-methylene cholesterol sulfate	10.09 ± 1.3
	Brassicasterol sulfate	3.91 ± 0.1
	Total StS	14.00 ± 1.45
*Diploneis* sp.	Brassicasterol sulfate	11.03 ± 2.9
	Total StS	11.03 ± 2.9

## Supplementary Materials

The following are available online at http://www.mdpi.com/1660-3397/17/1/10/s1, Table S1: Distribution of diatom species examined: comparison of StS retention time with deuterated standard (*m*/*z* 472.3) and synthetic derivatives, Figure S1–S6: Chromatograms of diatoms analyzed.
