# Supplementary material for: UPLC–MS/MS Identification of Sterol Sulfates in Marine Diatoms
Source: Mar Drugs. 2018 Dec 24;17(1):10. doi: 10.3390/md17010010 (PMC6356472; doi:10.3390/md17010010)
Supplement: Supplementary file 1 [file marinedrugs-17-00010-s001.pdf]

marine drugs

Supplementary Materials

UPLC–MS/MS Identification of Sterol Sulfates in Marine Diatoms

Genoveffa Nuzzo [1,*,†], Carmela Gallo [1,†], Giuliana d'Ippolito [1,†], Emiliano Manzo [1], Nadia Ruocco [1,2], Ennio Russo [2], Ylenia Carotenuto [2], Maria Costantini [2], Valerio Zupo [3], Angela Sardo [1] and Angelo Fontana [1]

[1] Bio-Organic Chemistry Unit, Institute of Biomolecular Chemistry CNR, Via Campi Flegrei 34, Pozzuoli, 80078 Naples, Italy; carmen.gallo@icb.cnr.it (C.G.); gdippolito@icb.cnr.it (G.I.); emanzo@icb.cnr.it (E.M.); nadia.ruocco@gmail.com (N.R.); angela.sardo@icb.cnr.it (A.S.)

[2] Department of Biology and Evolution of Marine Organisms, Stazione Zoologica Anton Dohrn, Villa Comunale, 80121 Napoli, Italy; ennio.erre@gmail.com (E.R.); ylenia@szn.it (Y.C.); mcosta@szn.it (M.C.)

[3] Center of Villa Dohrn Ischia-Benthic Ecology, Department of Integrative Marine Ecology, Stazione Zoologica Anton Dohrn, Via Porto 127, Ischia, 80077 Naples, Italy; vzupo@szn.it

† These authors have equally contributed to this work

* Correspondence: nuzzo.genoveffa@icb.cnr.it (G.N.)

marine drugs

MDPI

Table S1. Distribution of StS in diatom species examined: comparison of StS retention time with deuterated standard (CHOS-d = *m/z* 472.3) and synthetic derivatives.

Samples / *m/z* (StS)	C. scutellum	Diploneis sp.	N. Shiloi	C. closterum	C. cryptica	S. marinoi	*P. tricornutum*	*T. pseudonana*	*T. rotula*	Synthetic StS				
472.3 (CHOS-d)	5.24	5.41	5.27	5.22	5.20	5.16	4.96	5.05	5.02	5.21	5.25	5.26	5.25	5.24
463.3 (desmosterol sulfate)				4.80			4.68			4.82				
465.5 (cholesterol sulfate)						5.16								
477.3 (24-methylene cholesterol sulfate)	5.19				5.13	5.10	5.19	5.02	4.97					
477.3 (brassicasterol sulfate)	5.59	5.60	5.52							5.51				
479.3 (24-methyl cholesterol sulfate)					5.84	5.77		5.57	5.49		5.85			
491.3 (fucosterol sulfate)			5.76			5.80						5.82		
491.3 (stigmasterol sulfate)													6.28	
493.3 (-sitosterol sulfate)						6.33								6.45

marine drugs

MDPI

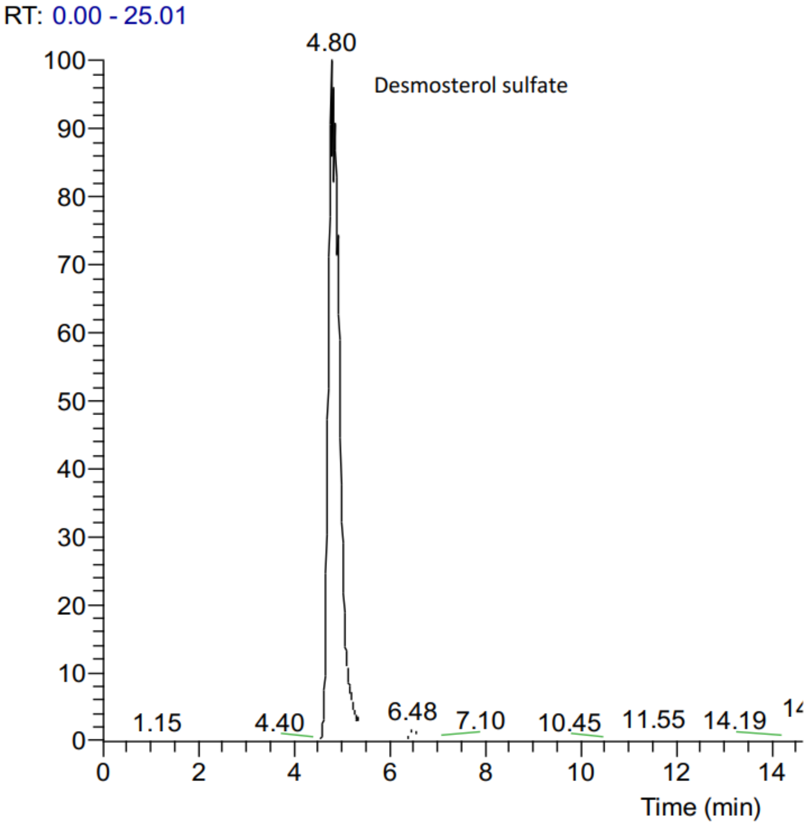

Figure S1. Chromatograms of StS content of *C. Closterum* (ion extract at *m*/*z* 463.3).

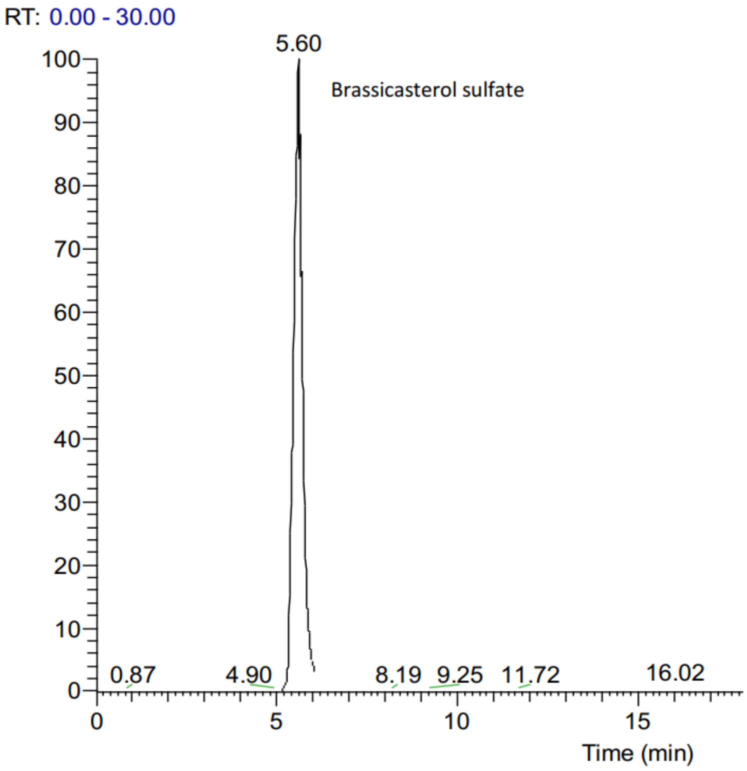

Figure S2. Chromatograms of StS content of *Diploneis sp.* (ion extract at *m*/*z* 477.3).

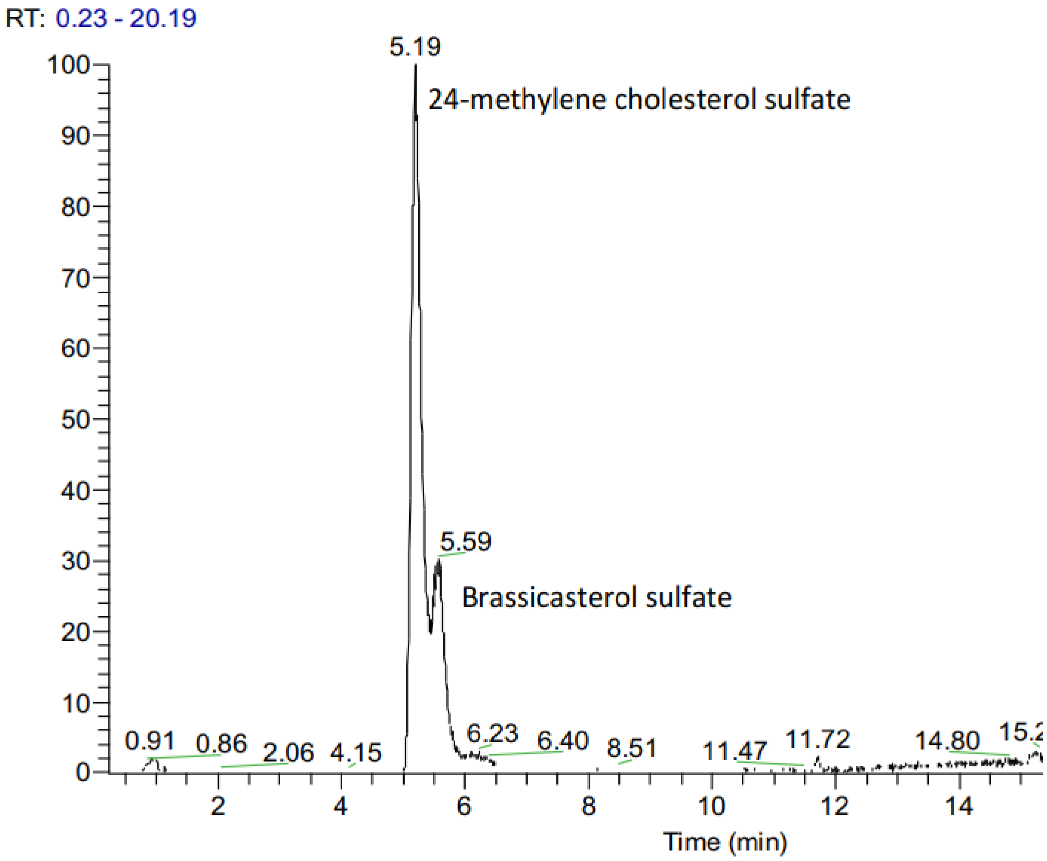

Figure S3. Chromatograms of StS content of *C. scutellum* (ion extract at *m*/*z* 477.3).

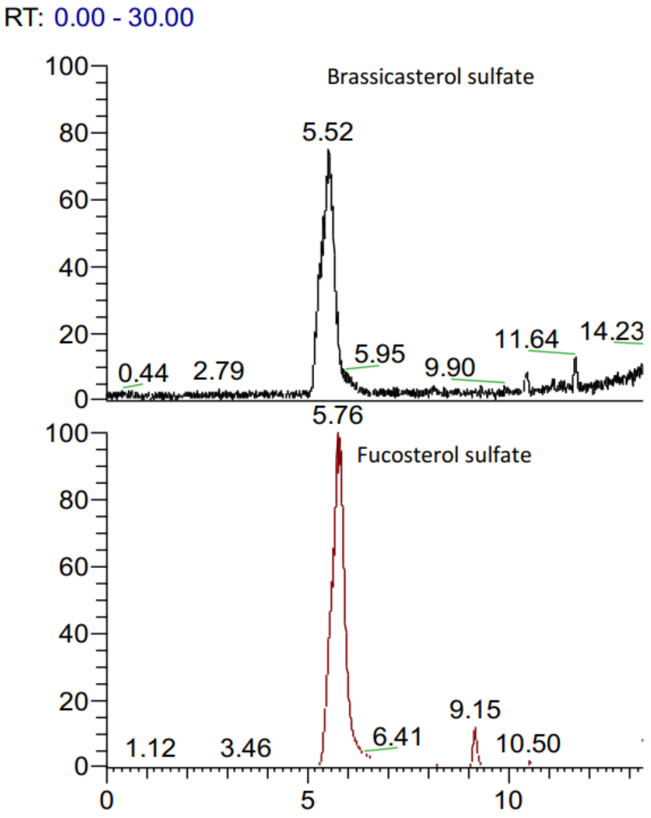

Figure S4. Chromatograms of StS content of *N. shiloi* (ion extract at *m*/*z* 477.3 and 491.3).

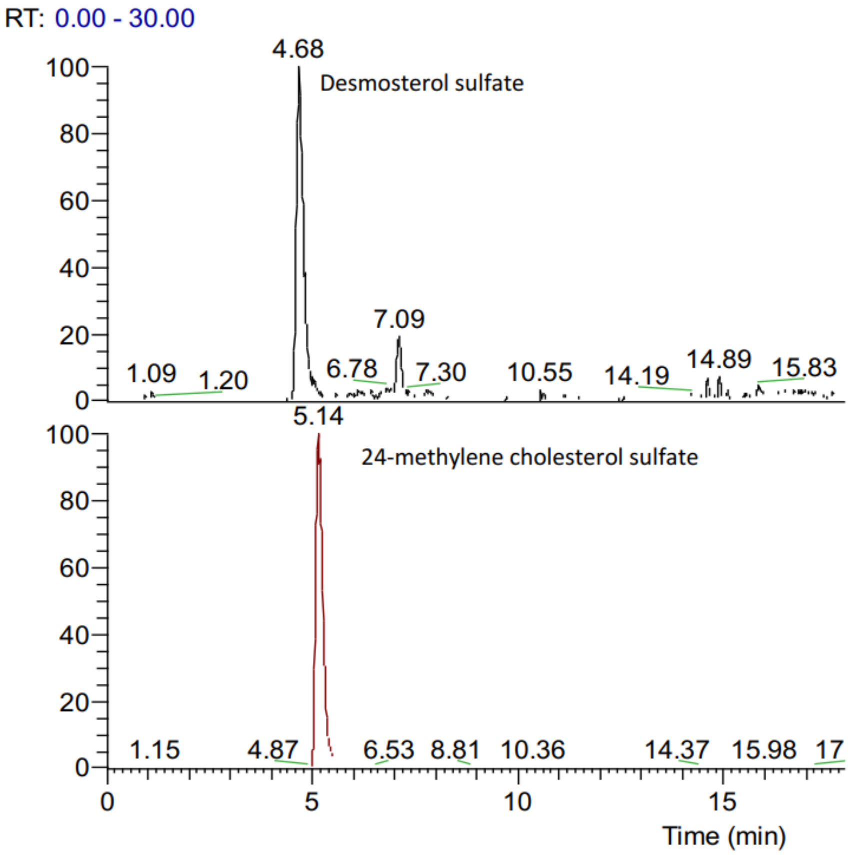

Figure S5. Chromatograms of StS content of *P.tricornutum* (ion extract at *m/z* 463.3 and 477.3).

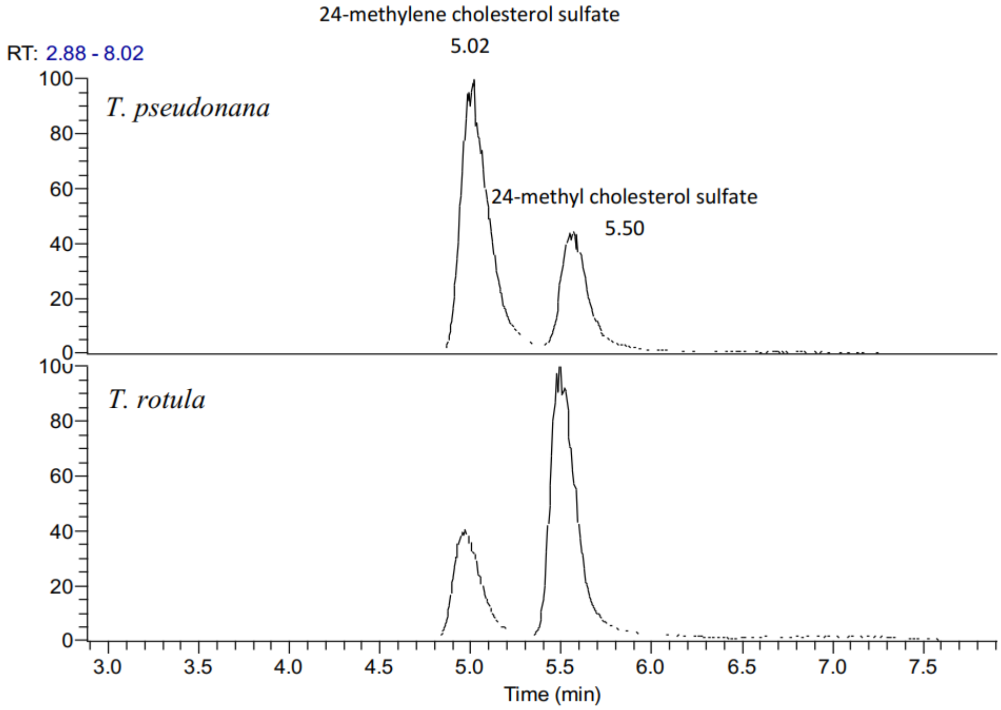

Figure S6. Chromatograms of StS content of *T. pseudonana e rotula* (ion extract at *m/z* 477.3 and 479.3).
